# Epulis

**Published:** 1851-07

**Authors:** 


*Epulis.*—Epulis of the gums, is too often temporized with, and allowed to increase or become malignant from the simple, small and benign tumor at its origin, by the use of escharotics and various inefficient styptics.

If the tumors originate between the teeth, it is more than probable, that they are carious, and are most frequently the cause, and as a general thing, one or both must be extracted. If not followed by removal of the trouble, the tumor must be cut away, and this is the proper treatment, under all circumstances, where extraction does not afford relief. It is generally considered to be caused by decayed teeth, diseased fangs and *bad operations in dentistry.*

Considering the fearful consequences this disease is occasionally attended with, prompt treatment becomes, in all cases, highly essential, and this treatment consists in excision of the affected part.

Mr. *Liston* expressed the opinion, that there was probably more surgery about the face and head, than in the rest of the body taken together, and many originating and depending on irritation and inflammation in the mouth or the organs within it, the superior and inferior maxillas and all other parts in connection, with them. The eye alone, is subject to one hundred and fifty-nine specific diseases, many requiring surgical operations of the first character. But this is principally owing to the constant exposure of this part of the body, and the aptitude of general diseases locating here as an effect.

